# Supplementary material for: Molecular characterization of haemagglutinin genes of influenza B viruses circulating in Ghana during 2016 and 2017
Source: PLoS One. 2022 Sep 23;17(9):e0271321. doi: 10.1371/journal.pone.0271321 (PMC9506629; doi:10.1371/journal.pone.0271321)
Supplement: S1 Fig — (PDF) [file pone.0271321.s001.pdf]

S1 Fig: A sample gel image

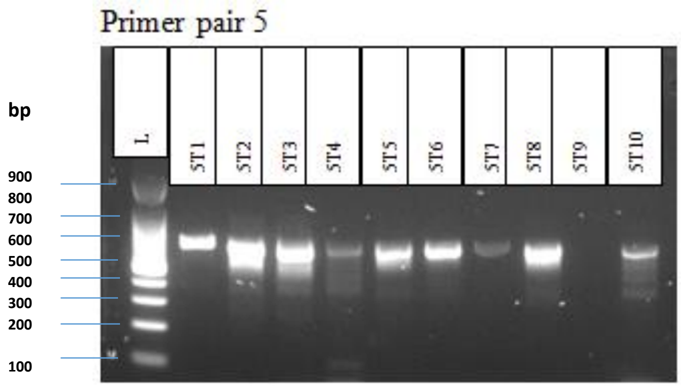

Legend: **L = 100bp Ladder**

5T1 – 5T8 = Primer 5, Sample/Template 1 – Primer pair 5, Sample/Template 8

5T9 = Primer pair 5, Negative Control

5T10 = Primer pair 5, Positive Control
