# Supplementary material for: Molecular characterization of haemagglutinin genes of influenza B viruses circulating in Ghana during 2016 and 2017
Source: PLoS One. 2022 Sep 23;17(9):e0271321. doi: 10.1371/journal.pone.0271321 (PMC9506629; doi:10.1371/journal.pone.0271321)
Supplement: S2 Fig — (PDF) [file pone.0271321.s002.pdf]

S2 Fig: HA amino acid alignment for Influenza B Victoria-Lineage viruses

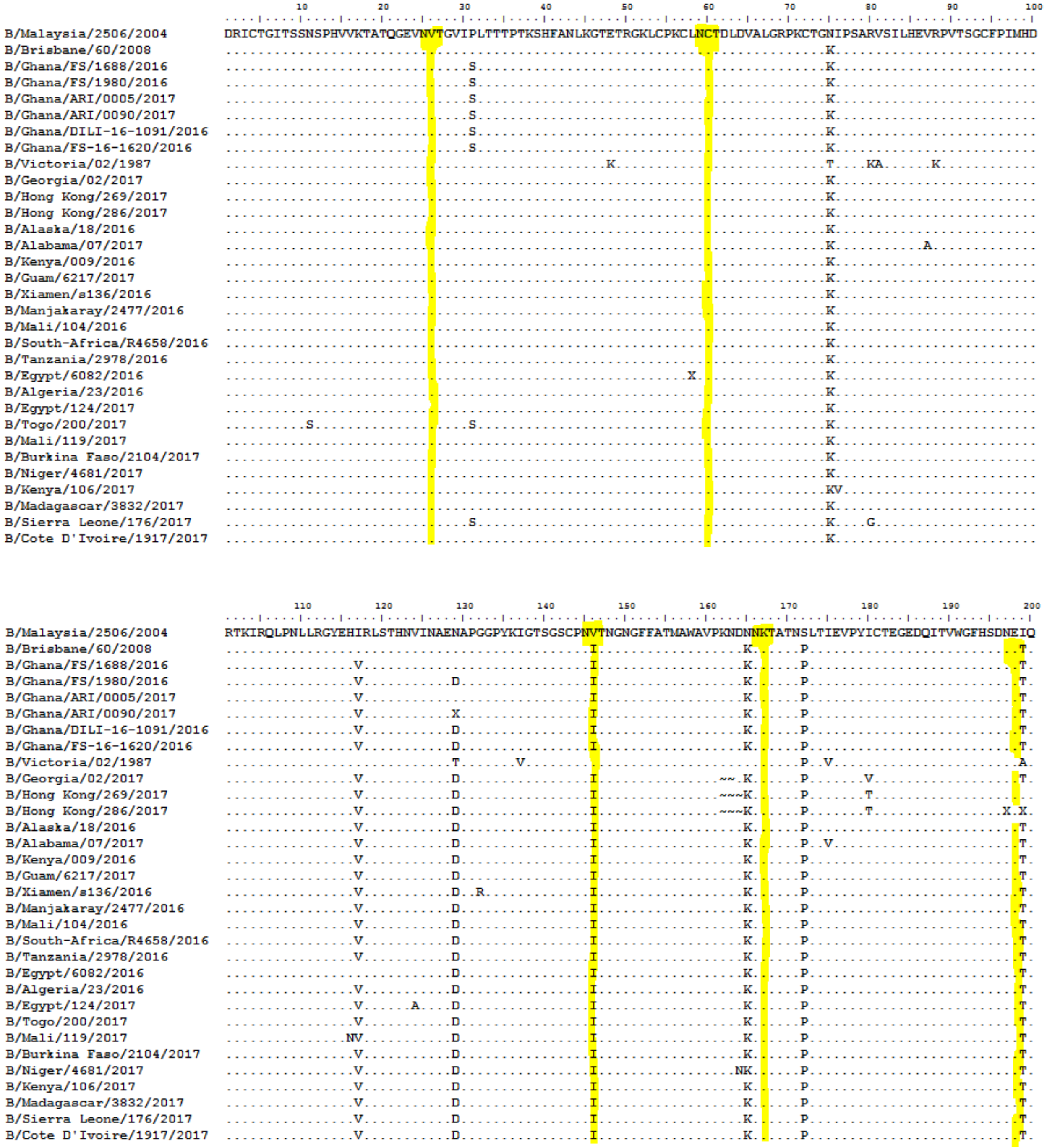

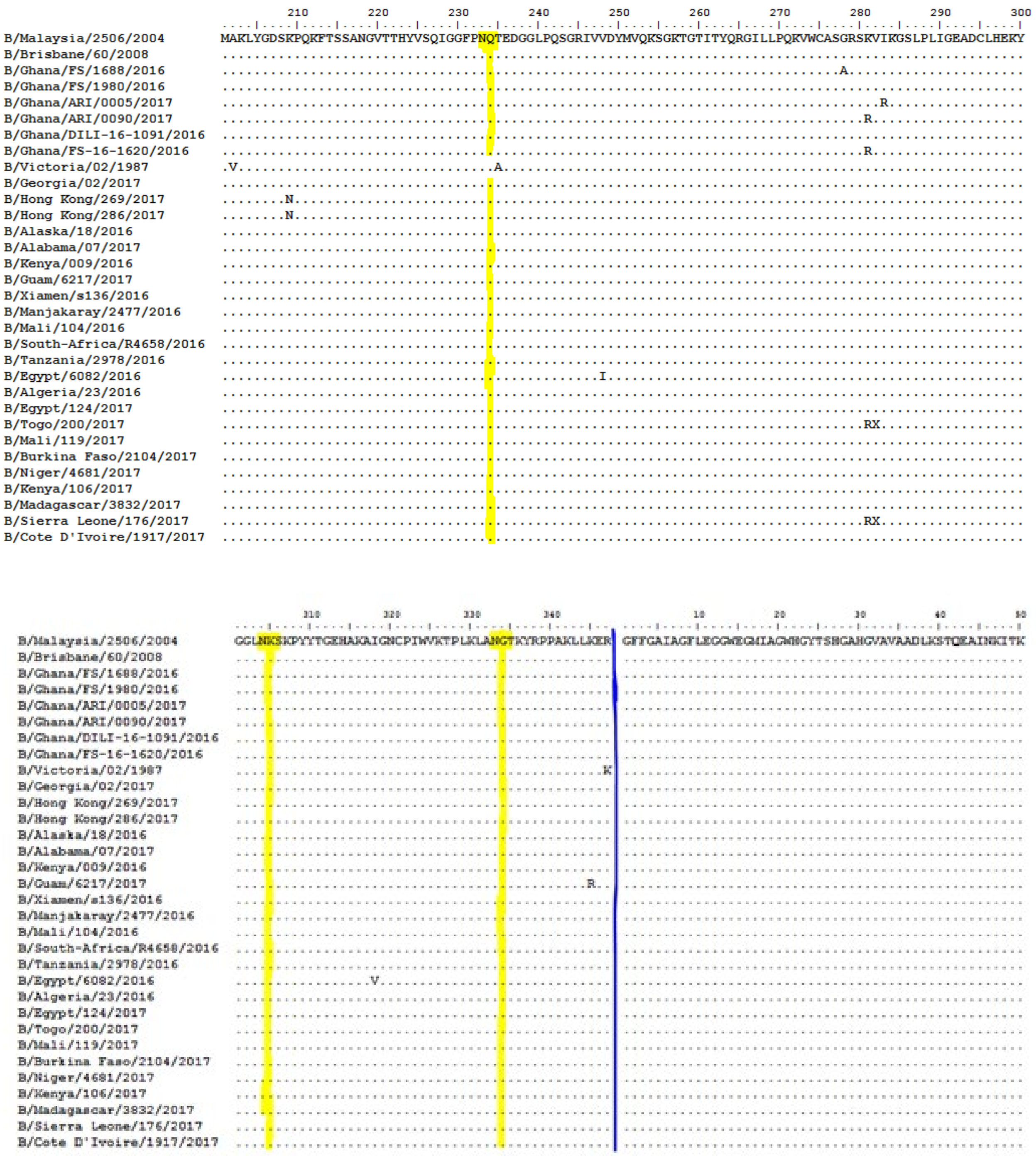
210 220 230 240 250 260 270 280 290 300
B/Malaysia/2506/2004 MAKLYGDSKPQKFTSSANGVTTHYVSQIGGFPNQTEDGGLPQSGRIVVDYMVQKSGKTGTITYQRGILLPQKVWCASGRSKVIKGSLPLIGEADCLHEKY
B/Brisbane/60/2008
B/Ghana/FS/1688/2016
B/Ghana/FS/1980/2016
B/Ghana/ARI/0005/2017
B/Ghana/ARI/0090/2017
B/Ghana/DILI-16-1091/2016
B/Ghana/FS-16-1620/2016
B/Victoria/02/1987
B/Georgia/02/2017
B/Hong Kong/269/2017
B/Hong Kong/286/2017
B/Alaska/18/2016
B/Alabama/07/2017
B/Kenya/009/2016
B/Guam/6217/2017
B/Xiamen/s136/2016
B/Manjakaray/2477/2016
B/Mali/104/2016
B/South-Africa/R4658/2016
B/Tanzania/2978/2016
B/Egypt/6082/2016
B/Algeria/23/2016
B/Egypt/124/2017
B/Togo/200/2017
B/Mali/119/2017
B/Burkina Faso/2104/2017
B/Niger/4681/2017
B/Kenya/106/2017
B/Madagascar/3832/2017
B/Sierra Leone/176/2017
B/Cote D'Ivoire/1917/2017
310 320 330 340 10 20 30 40 50
B/Malaysia/2506/2004 GGLNKSKPYYTGEHAKAIGNCPIWVKTPLKLANGTKYRPPAKLLKER GFFGAIAGFLEGGWEGMIAGWHGYTSHGAHGVAVAADLKSTQEAINKITK

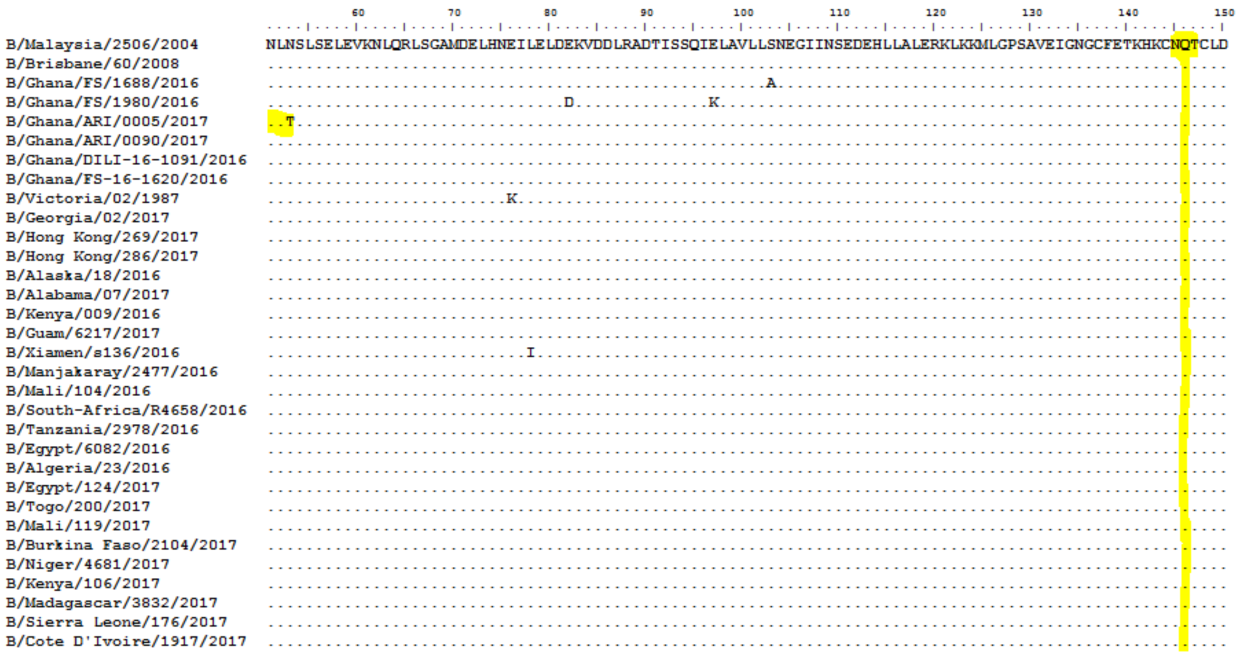

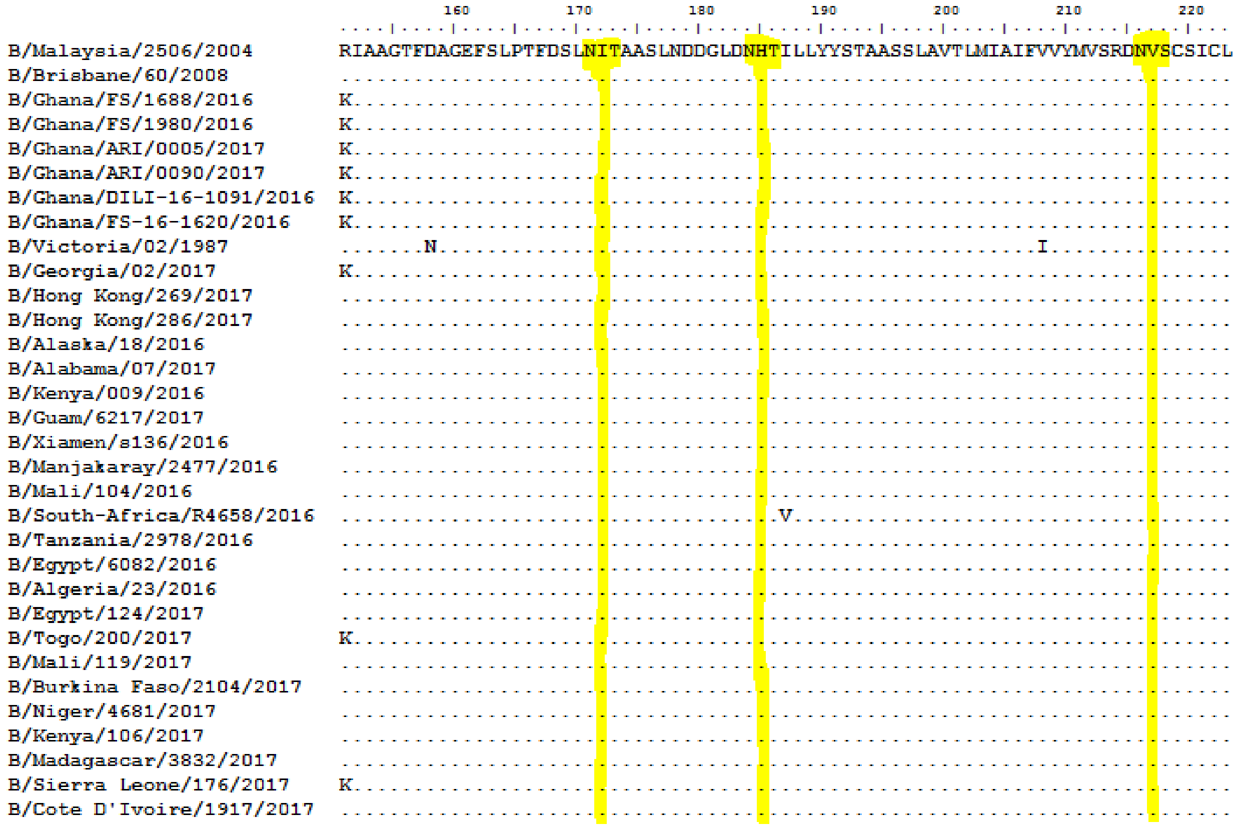

Note: The highlighted yellow shows the amino acid positions for potential N-linked glycosylation sites. The blue highlight indicates the HA1/HA2 boundary.
