# Supplementary material for: Molecular characterization of haemagglutinin genes of influenza B viruses circulating in Ghana during 2016 and 2017
Source: PLoS One. 2022 Sep 23;17(9):e0271321. doi: 10.1371/journal.pone.0271321 (PMC9506629; doi:10.1371/journal.pone.0271321)
Supplement: S3 Fig — (PDF) [file pone.0271321.s003.pdf]

S3 Fig: HA amino acid alignment for Influenza B Yamagata-Lineage

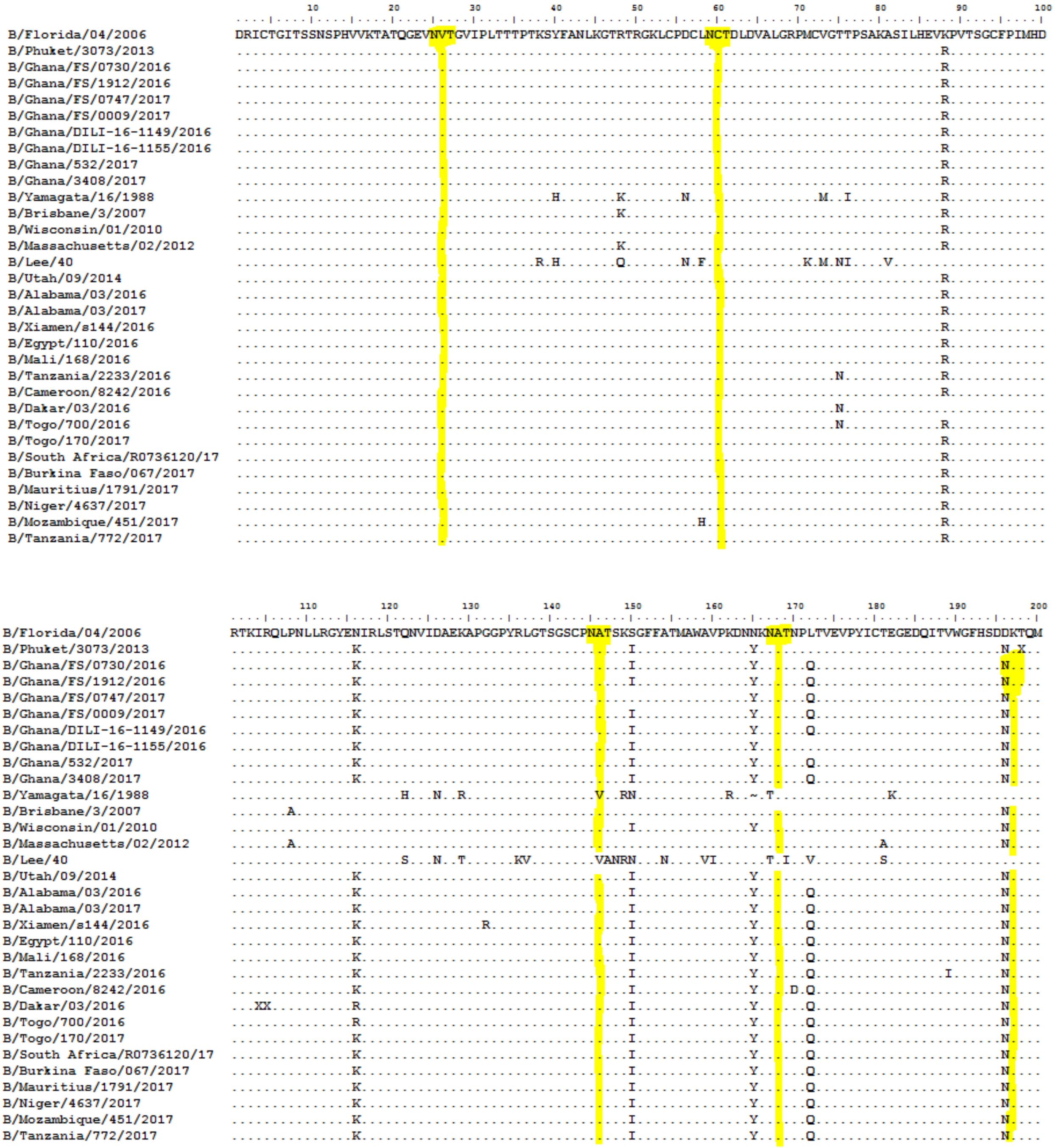

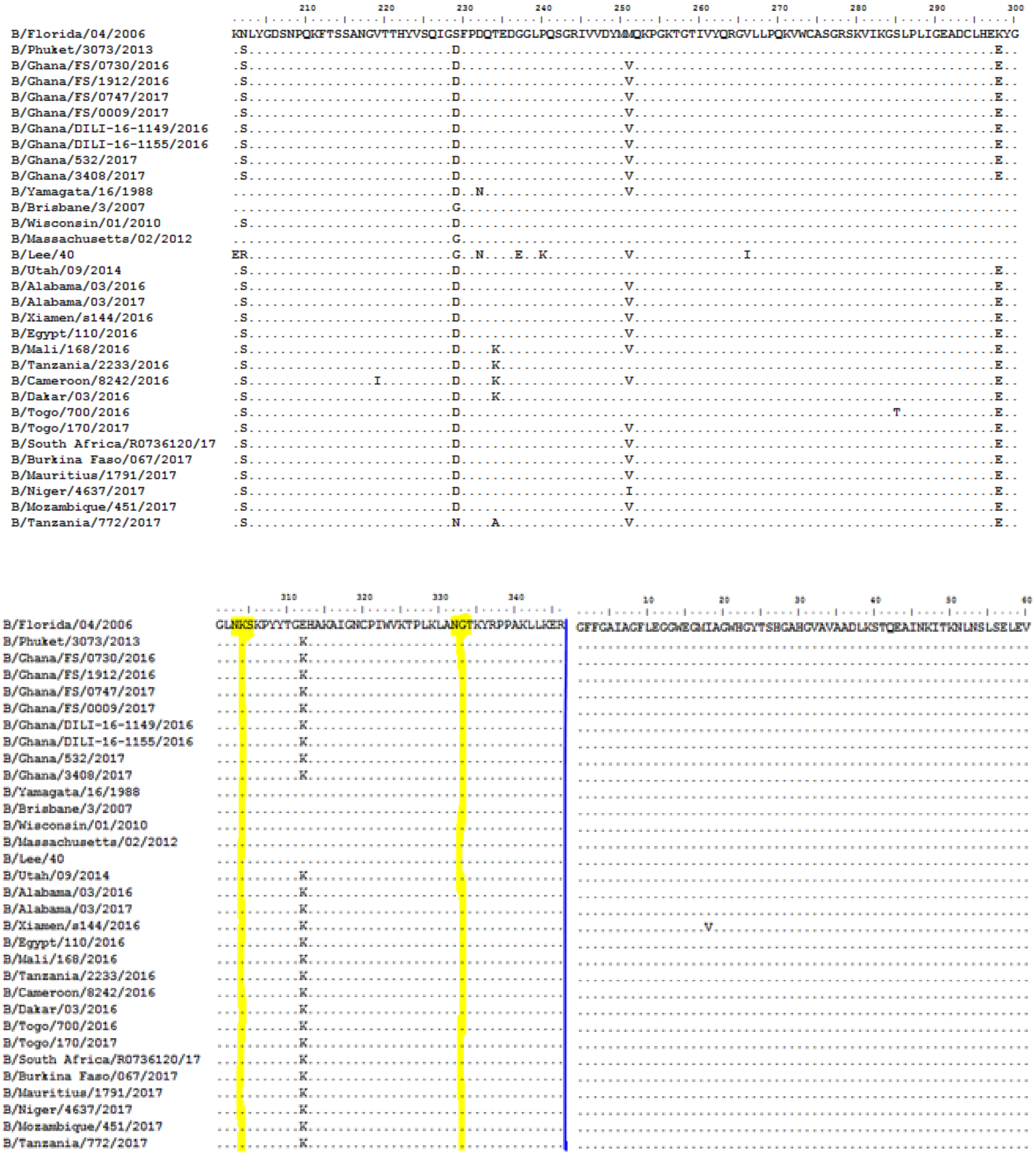
210 220 230 240 250 260 270 280 290 300
B/Florida/04/2006
KNLYGDSNPQKFTSSANGVTTHYVSQIGSFPDQTEDGGLPQSGRIVVDYMMQKPGKTGTIVYQRGVLLPQKVWCASGRSKVIKGSLPLIGEADCLHEKYG
B/Phuket/3073/2013
B/Ghana/FS/0730/2016
B/Ghana/FS/1912/2016
B/Ghana/FS/0747/2017
B/Ghana/FS/0009/2017
B/Ghana/DILI-16-1149/2016
B/Ghana/DILI-16-1155/2016
B/Ghana/532/2017
B/Ghana/3408/2017
B/Yamagata/16/1988
B/Brisbane/3/2007
B/Wisconsin/01/2010
B/Massachusetts/02/2012
B/Lee/40
B/Utah/09/2014
B/Alabama/03/2016
B/Alabama/03/2017
B/Xiamen/s144/2016
B/Egypt/110/2016
B/Mali/168/2016
B/Tanzania/2233/2016
B/Cameroon/8242/2016
B/Dakar/03/2016
B/Togo/700/2016
B/Togo/170/2017
B/South Africa/R0736120/17
B/Burkina Faso/067/2017
B/Mauritius/1791/2017
B/Niger/4637/2017
B/Mozambique/451/2017
B/Tanzania/772/2017
310 320 330 340
10 20 30 40 50 60
GLNKSKPYYTGEHAKAIGNCPIWVKTPLKLANGTKYRPPAKLLKER
GFFGAIAGFLEGGWEGMIAGWHGYTSHGAHGVAVAADLKSTQEAINKITKNLNSLSELEV

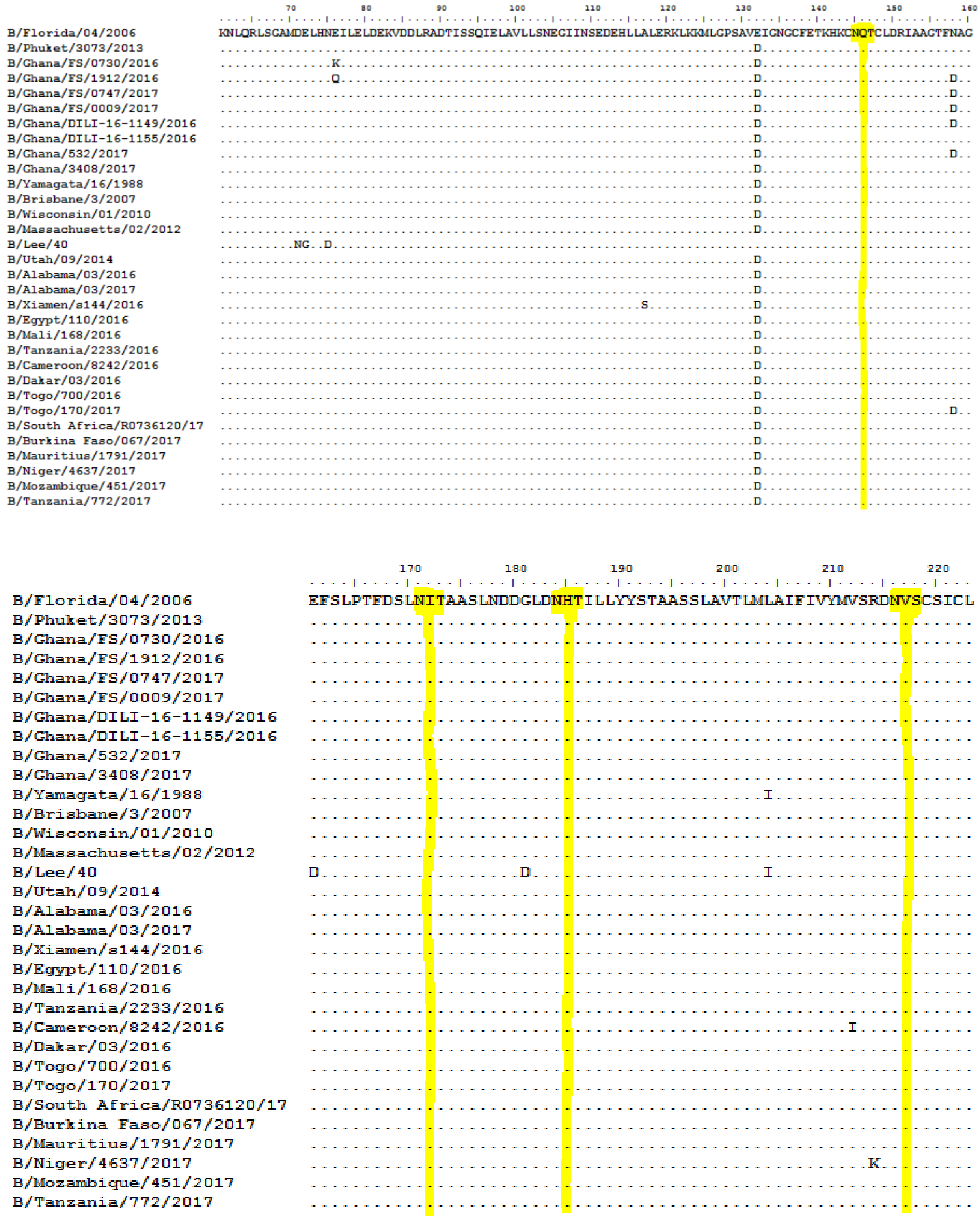

Note: The highlighted yellow shows the amino acid positions for potential N-linked glycosylation sites. The blue highlight indicates the HA1/HA2 boundary.
