# Supplementary material for: Molecular characterization of haemagglutinin genes of influenza B viruses circulating in Ghana during 2016 and 2017
Source: PLoS One. 2022 Sep 23;17(9):e0271321. doi: 10.1371/journal.pone.0271321 (PMC9506629; doi:10.1371/journal.pone.0271321)
Supplement: S1 Table — (PDF) [file pone.0271321.s004.pdf]

S1 Table: Details of primers used for this research

PRIMER ID	PRIMER SEQUENCES (5' - 3')	LOCATION ON HA GENE
BHAF1U*	TATTCGTCTCAGGGAGCAGAAGCAG	1-27
BHA1F1 (BHAF22)	AATATCCACAAAATGAAGGCAATA	22-45
BHAF355-12	GGGTGCTTYCCTATAATGCACGA	355-377
BHAF458	AGAAAAGGCACCAGGAGGACCCTA	459-482
BHAF880-12	TTRTTGCCTCAAAAGGTGTGGTG	880-902
BHAF1432-12	GAAGGAATAATAAACAGTGAAGA	1432-1454
RBHAF1435	GGAATAATAAACAGTGAAGAYGAGCA	1435-1460
BHAR552-12	CCAAGCCATTGTTGCGAARAATCC	552-529
BHAR918-12	GCTCCTGCCACTYGCGCACCA	918-898
BHAR962	TGGAGGCAATCTGCTTCACC	962-943
BHAR1341	TTCGTTGTGGAGTTCATCCAT	1347-1327
RBHAR1492	TCTACAGCAGAGGGRCCCAGGAT	1514-1492
BHA2R1	GTAATGGTAACAAGCAAACAAGCA	1849-1826

*-Primer sequences have been modified (previous length was 41bases).

Source: [26, 27].
