# Supplementary material for: Molecular characterization of haemagglutinin genes of influenza B viruses circulating in Ghana during 2016 and 2017
Source: PLoS One. 2022 Sep 23;17(9):e0271321. doi: 10.1371/journal.pone.0271321 (PMC9506629; doi:10.1371/journal.pone.0271321)
Supplement: S2 Table — (PDF) [file pone.0271321.s005.pdf]

S2 Table: Amino acid substitutions in the HA genes of the study sequences, compared to the Influenza B Victoria reference strain B/Brisbane/60/2008

Virus Strains	HA1 Amino acid at position					HA2 Amino acid at position					
	**31**	**117**	**129**	**278**	**283**	**53**	**82**	**92**	**97**	**103**	**151**
**B/Brisbane/60/2008**	**P**	**I**	**N**	**G**	**I**	**N**	**E**	**I**	**E**	**S**	**R**
B/Ghana/FS/1688/2016	**S**	**V**	-	**A**	-	-	-	**K**	-	**A**	**K**
B/Ghana/FS/1980/2016	**S**	**V**	**D**	-	-	-	**D**	-	**R**	-	**K**
B/Ghana/ARI/0005/2017	**S**	**V**	-	-	**R**	**T**	-	-	-	-	**K**
B/Ghana/ARI/0090/2017	**S**	**V**	-	-	-	-	-	-	-	-	**K**
B/Ghana/DILI-16-1091/2016	**S**	**V**	**D**	-	-	-	-	-	-	-	**K**
B/Ghana/FS-16-1620/2016	**S**	**V**	**D**	-	-	-	-	-	-	-	**K**

Key: S (Serine), P (Proline), I (Isoleucine), V (Valine), N (Asparagine), D (Aspartic acid), G (Glycine), A (Alanine), K (Lysine), R (Arginine), T (Threonine), E (Glutamic acid), - (Consensus to reference sequence).
