# Supplementary material for: Molecular characterization of haemagglutinin genes of influenza B viruses circulating in Ghana during 2016 and 2017
Source: PLoS One. 2022 Sep 23;17(9):e0271321. doi: 10.1371/journal.pone.0271321 (PMC9506629; doi:10.1371/journal.pone.0271321)
Supplement: S3 Table — (PDF) [file pone.0271321.s006.pdf]

S3 Table: Amino acid substitutions in the HA genes of the study sequences, compared to the influenza B Yamagata reference strain B/Phuket/3073/2013

**Virus Strains**	**HA1 Amino acid at position**				**HA2 Amino acid at position**	
	**150**	**172**	**198**	**251**	**76**	**158**
**B/Phuket/3073/2013**	**I**	**L**	**TI**	**M**	**E**	**N**
B/Ghana/FS/0730/2016	**-**	**Q**	**-**	**V**	**K**	**-**
B/Ghana/FS/1912/2016	**-**	**Q**	**-**	**V**	**Q**	**D**
B/Ghana/FS/0747/2017	**S**	**Q**	**-**	**V**	**-**	**D**
B/Ghana/FS/0009/2017	**-**	**Q**	**-**	**V**	**-**	**D**
B/Ghana/DILI-16-11149-2016	**-**	**Q**	**-**	**V**	**-**	**D**
B/Ghana/532/2017	**-**	**Q**	**-**	**V**	**-**	**D**

Key: S (Serine), I (Isoleucine), V (Valine), N (Asparagine), D (Aspartic acid), K (Lysine), T (Threonine), E (Glutamic acid), Q (Glutamine), L (Leucine), M (Methionine), - (Consensus to reference sequence). At position 198, the study and the Ghanaian reference sequences have T.
