# Supplementary material for: Molecular characterization of haemagglutinin genes of influenza B viruses circulating in Ghana during 2016 and 2017
Source: PLoS One. 2022 Sep 23;17(9):e0271321. doi: 10.1371/journal.pone.0271321 (PMC9506629; doi:10.1371/journal.pone.0271321)
Supplement: S4 Table — (PDF) [file pone.0271321.s007.pdf]

S4 Table. Accession numbers of influenza B sequences in NCBI influenza Virus Resource and GISAID used for the phylogenetic reconstruction of HA genes.

Strains	Collection Date	Accession Numbers for HA genes	Status
B/Malaysia/2506/2004	2004	CY038287	Reference
B/Victoria/02/1987	1982	CY018757	Reference
B/Brisbane/60/2008	04-08-2008	FJ766840	WHO Vaccine
B/Egypt/6082/2016	17-07-2016	EPI237678	Reference
B/Alaska/18/2016	29-11-2016	CY218283	Reference
B/Tanzania/2978/2016	23-06-2016	EPI235482	Reference
B/Guam/6217/2017	23-09-2017	CY244329	Reference
B/Kenya/009/2016	01-03-2016	KY210889	Reference
B/Alabama/07/2017	13-05-2017	CY240189	Reference
B/Kenya/106/2017	22-07-2017	EPI287641	Reference
B/Togo/200/2017	04-03-2017	EPI277263	Reference
B/Ghana/FS-16-1620/2016	26-10-2016	EPI248675	Reference
B/Ghana/DILI-16-1091/2016	26-10-2016	EPI248671	Reference
B/Sierra-Leone/176/2017	10-10-2017	EPI299864	Reference
B/Georgia/02/2017	11-01-2017	EPI255229	Reference
B/South- Africa/R4658/2016	05-07-2016	EPI232919	Reference
B/Mali/104/2016	09-03-2016	EPI232277	Reference

Strains	Collection Date	Accession Numbers for HA genes	Status
B/Burkina Faso/2104/2017	01-03-2016	EPI284974	Reference
B/Egypt/124/2017	04-01-2017	EPI269525	Reference
B/Code D'ivoire/1917/2017	06-11-2017	EPI300282	Reference
B/Niger/4681/2017	22-02-2017	EPI287095	Reference
B/Mali/119/2017	22-05-2017	EPI281676	Reference
B/Madagascar/3832/2017	04-09-2017	EPI296534	Reference
B/Manjakaray/2477/2016	01-06-2016	EPI231720	Reference
B/Xiamen/s136/2016	01-04-2016	KY273068	Reference
B/Algeria/23/2016	28-11-2016	EPI261207	Reference
B/Hong-Kong/286/2017	06-06-2017	EPI290862	Reference
B/Hong-Kong/269/2017	2017	EPI291287	Reference
B/Lee/40	1940	DQ792897	Reference
B/Yamagata/16/1988	1988	M58419	Reference
B/Florida/04/2016	01-11-2016	EU100604	Reference
B/Massachusetts/02/2012	13-03-2012	KC891816	WHO Vaccine
B/Brisbane/3/2007	09-03-2007	KP460690	WHO Vaccine
B/Wisconsin/01/2010	20-02-2010	JN993031	WHO Vaccine
B/Ghana/DILI-16-1155/2016	10-11-2016	EPI248673	Reference
B/Utah/09/2014	29-05-2014	KU592766	WHO Vaccine

Strains	Collection Date	Accession Numbers for HA genes	Status
B/Phuket/3073/2013	21-11-2013	EPI186619	WHO Vaccine
B/Niger/4637/2017	20-02-2017	EPI287043	Reference
B/Dakar/03/2016	09-11-2016	EPI261237	Reference
B/Tanzania/2233/2016	12-04-2016	EPI235489	Reference
B/Togo/700/2016	17-12-2016	EPI277251	Reference
B/Cameroon/8242/2016	30-09-2016	EPI261235	Reference
B/Mali/168/2016	25-04-2016	EPI235002	Reference
B/Burkina Faso/067/2017	11-01-2017	EPI284924	Reference
B/Egypt/110/2016	06-03-2016	EPI224392	Reference
B/Alabama/03/2016	13-12-2016	CY236446	Reference
B/Togo/170/2017	18-02-2017	EPI277262	Reference
B/Ghana/DILI-16-1149/2016	09-11-2016	EPI248672	Reference
B/Ghana/532/2017	28-02-2017	EPI277072	Reference
B/Xiamen/s144/2016	18-02-2016	KY273076	Reference
B/Alabama/03/2017	20-02-2017	CY224518	Reference
B/Tanzania/772/2017	22-12-2017	EPI298462	Reference
B/Mozambique/451/2017	02-11-2017	EPI292692	Reference
B/Ghana/3408/2017	08-11-2017	EPI291558	Reference
B/Mauritius/179/2017	20-09-2017	EPI287040	Reference

Strains	Collection Date	Accession Numbers for HA genes	Status
B/South-Africa/R0736120/17	25-07-2017	EPI282358	Reference
B/Ghana/FS/1688/2016	03-11-2016	MH748708	Ghanaian
B/Ghana/FS/1980/2016	14-12-2016	MH748709	Ghanaian
B/Ghana/ARI/0005/2017	12-01-2017	MH748710	Ghanaian
B/Ghana/ARI/0090/2017	10-02-2017	MH748711	Ghanaian
B/Ghana/FS/0730/2016	02-06-2016	MH748712	Ghanaian
B/Ghana/FS/1912/2016	29-12-2016	MH748713	Ghanaian
B/Ghana/FS/0747/2017	20-03-2017	MH748714	Ghanaian
B/Ghana/FS/0009/2017	04-01-2017	MH748715	Ghanaian
