# Supplementary material for: Molecular characterization of haemagglutinin genes of influenza B viruses circulating in Ghana during 2016 and 2017
Source: PLoS One. 2022 Sep 23;17(9):e0271321. doi: 10.1371/journal.pone.0271321 (PMC9506629; doi:10.1371/journal.pone.0271321)
Supplement: S5 Table — (PDF) [file pone.0271321.s008.pdf]

S5 Table: Potential Glycosylation sites of influenza B Victoria HA genes

Strain	Number of glycosylation sites	HA1 Amino acid position								HA2 Amino acid position				
		25	59	145	166	197	233	304	333	51	145	171	184	216
**B/Brisbane/60/2008**	12	NVT	NCT	NVT	NKT	NET	NQT	NKS	NCT	NLN	NQT	NIT	NHT	NVS
***B/Ghana/FS/1688/2016**	12	-	-	-	-	-	-	-	-	-	-	-	-	-
***B/Ghana/FS/1980/2016**	12	-	-	-	-	-	-	-	-	-	-	-	-	-
***B/Ghana/ARI/0005/2017**	13	-	-	-	-	-	-	-	-	NLT	-	-	-	-
***B/Ghana/ARI/0090/2017**	12	-	-	-	-	-	-	-	-	-	-	-	-	-
**B/Ghana/DILI-16-1091/2016**	12	-	-	-	-	-	-	-	-	-	-	-	-	-
**B/Ghana/FS-16-1620/2016**	12	-	-	-	-	-	-	-	-	-	-	-	-	-

Key: N (Asparagine), V (Valine), T (Threonine), C (Cysteine), K (Lysine), E (Glutamic acid), Q (Glutamine), S (Serine), L (Leucine), I (Isoleucine), H (Histidine), - (Consensus), * (sequences obtained from this study).
