# Supplementary material for: Molecular characterization of haemagglutinin genes of influenza B viruses circulating in Ghana during 2016 and 2017
Source: PLoS One. 2022 Sep 23;17(9):e0271321. doi: 10.1371/journal.pone.0271321 (PMC9506629; doi:10.1371/journal.pone.0271321)
Supplement: S6 Table — (PDF) [file pone.0271321.s009.pdf]

S6 Table: Potential Glycosylation sites of Influenza B Yamagata HA genes

Strain	Number of glycosylation sites	HA1 Amino acid position							HA2 amino acid position			
		25	59	145	167	196	303	332	145	171	184	216
**B/Phuket/3073/2013**	**10/11**	NVT	NCT	NAT	NAT	NKT (I)	NKS	NCT	NQT	NIT	NHT	NVS
***B/Ghana/FS/0730/2016**	11	-	-	-	-	NKT	-	-	-	-	-	-
***B/Ghana/FS/1912/2016**	11	-	-	-	-	NKT	-	-	-	-	-	-
***B/Ghana/FS/0747/2017**	11	-	-	-	-	NKT	-	-	-	-	-	-
***B/Ghana/FS/0009/2017**	11	-	-	-	-	NKT	-	-	-	-	-	-
**B/Ghana/DILI-16-11149-2016**	11	-	-	-	-	NKT	-	-	-	-	-	-
**B/Ghana/532/2017**	11	-	-	-	-	NKT	-	-	-	-	-	-

Key: N (Asparagine), V (Valine), T (Threonine), C (Cysteine), A (Alanine), K (Lysine), I (Isoleucine), S (Serine), Q (Glutamine), H (Histidine), - (Consensus). The vaccine sequences had amino acid degeneracy (T/I) at position 198. If T is present, glycosylation will be possible otherwise with I, no glycosylation, * (sequences obtained from this study)
