# Supplementary material for: Molecular characterization of haemagglutinin genes of influenza B viruses circulating in Ghana during 2016 and 2017
Source: PLoS One. 2022 Sep 23;17(9):e0271321. doi: 10.1371/journal.pone.0271321 (PMC9506629; doi:10.1371/journal.pone.0271321)
Supplement: S1 Raw images — (PDF) [file pone.0271321.s010.pdf]

S1_raw_images

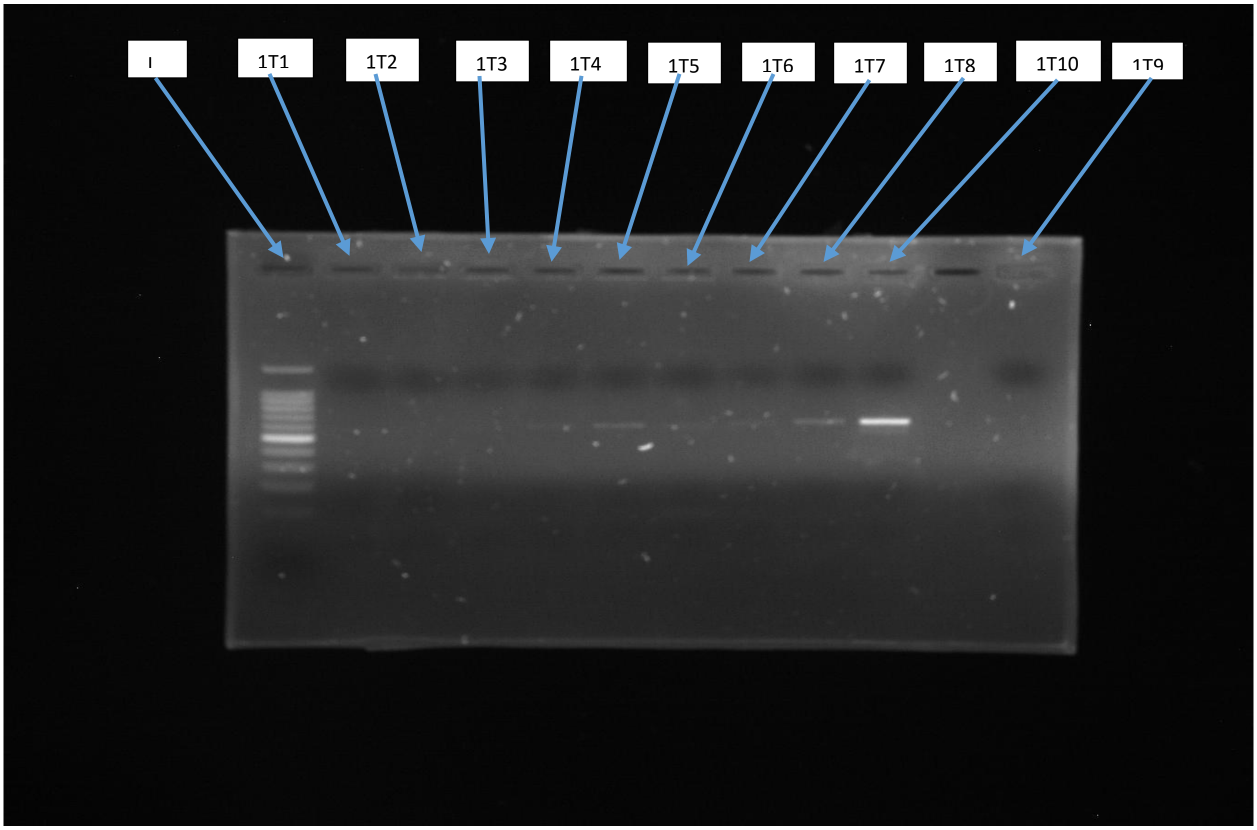

Gel image 1 (525bp): 1T1, 1T2, 1T3 and 1T4 were repeated

Key: T1 (B/Ghana/FS/1688/2016), T2 (B/Ghana/FS/1980/2016), T3 (B/Ghana/ARI/0005/2017) and T4 (B/Ghana/ARI/0090/2017) all influenza B Victoria lineage. T5 (B/Ghana/FS/0730/2016), T6 (B/Ghana/FS/1912/2016), T7 (B/Ghana/FS/0747/2017) and T8 (B/Ghana/FS/0009/2017) all influenza B Yamagata lineage. L represents a 100bp ladder (molecular weight marker), T9 (Negative control) and T10 (Positive control). Only gel image 5 was mentioned in the paper as the sample gel image. Each sample was sub-divided in to 8 fragments (eg 1T1, 2T1, 3T1, 4T1, 5T1, 6T1, 7T1 and 8T1) making a total of 64 fragments. Most fragments amplification were only successful after second or third attempts. In all, 52 amplification fragments were purified for sequencing. However, the overlapping nature of the fragments made room for the remaining 12 fragments that were not amplified to be covered. An Agarose gel of concentration 1.5% was prepared in 1X Tris-acetate EDTA (TAE) buffer. A 100bp DNA ladder (Promega., Switzerland) was loaded alongside amplicons to aid in the confirmation of the expected sizes. The gel was visualized using UV-Transilluminator (BioDoc-it^{TM} 220 imaging system; Upland, CA, USA). This key note is applicable to all other gel images within this file.

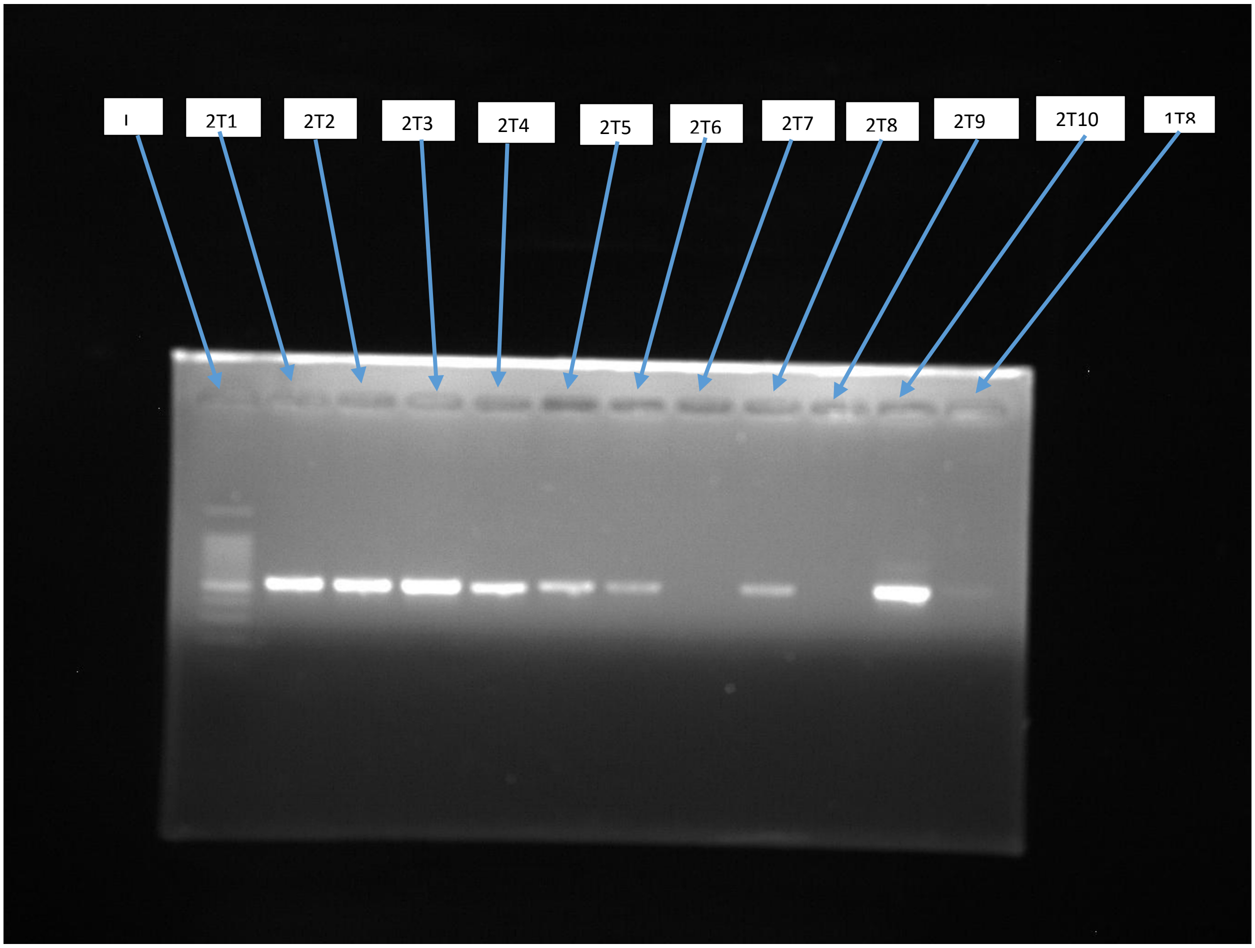

Gel image 2 (498bp): All fragments amplified except 2T7 and 1T8

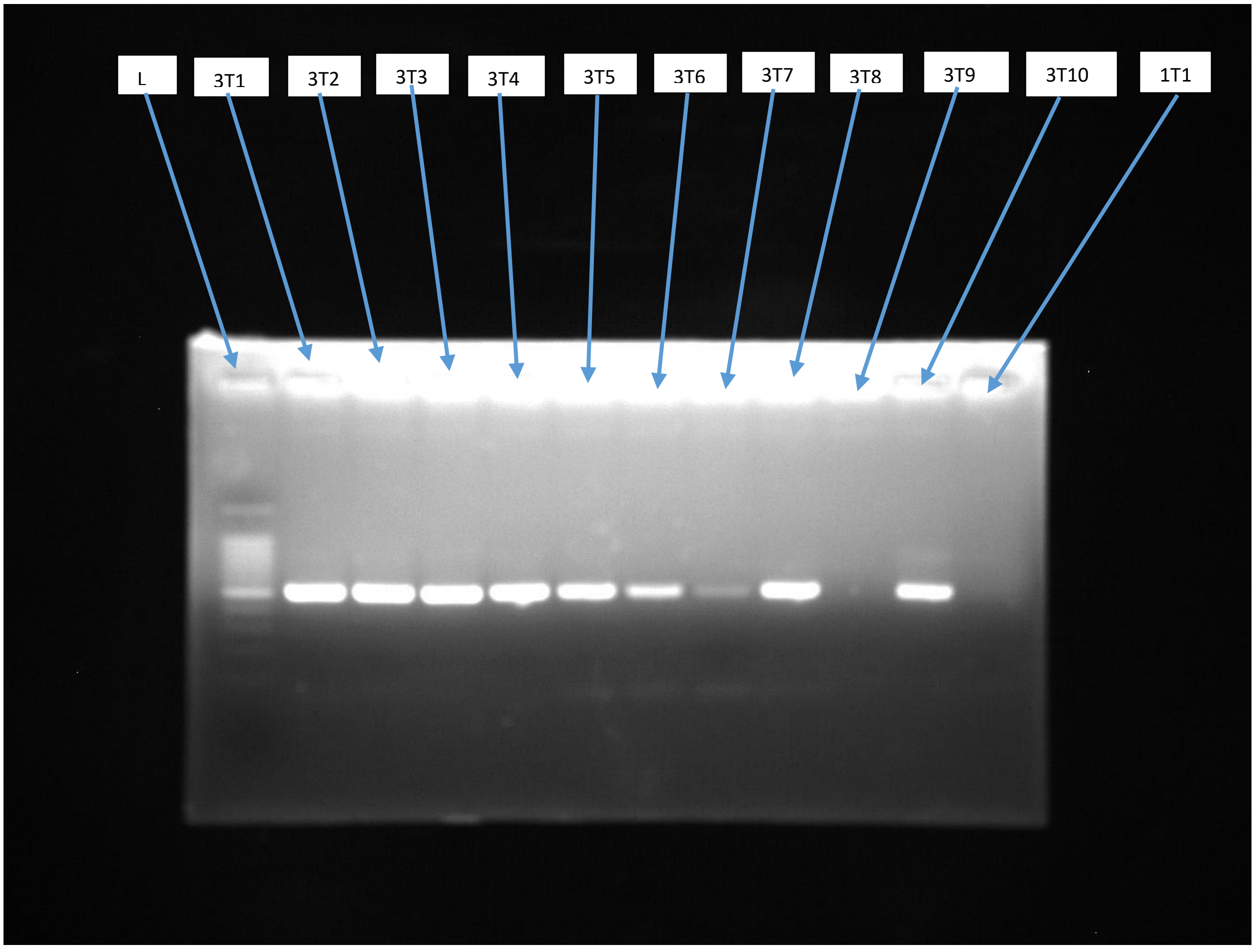

Gel image 3 (480bp): All fragments amplified except 1T1

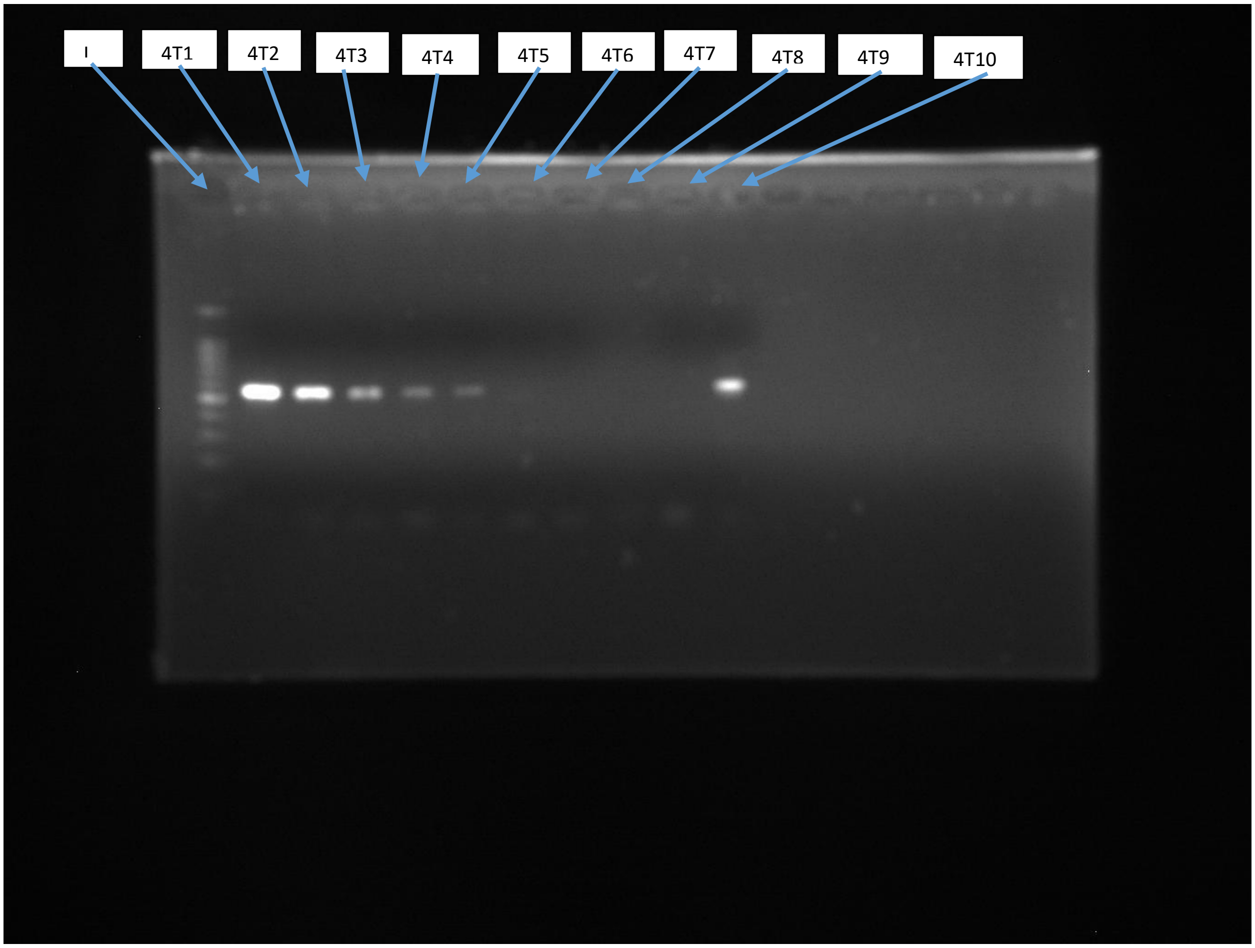

Gel image 4 (541bp): No amplification band for 4T6, 4T7 and 4T8

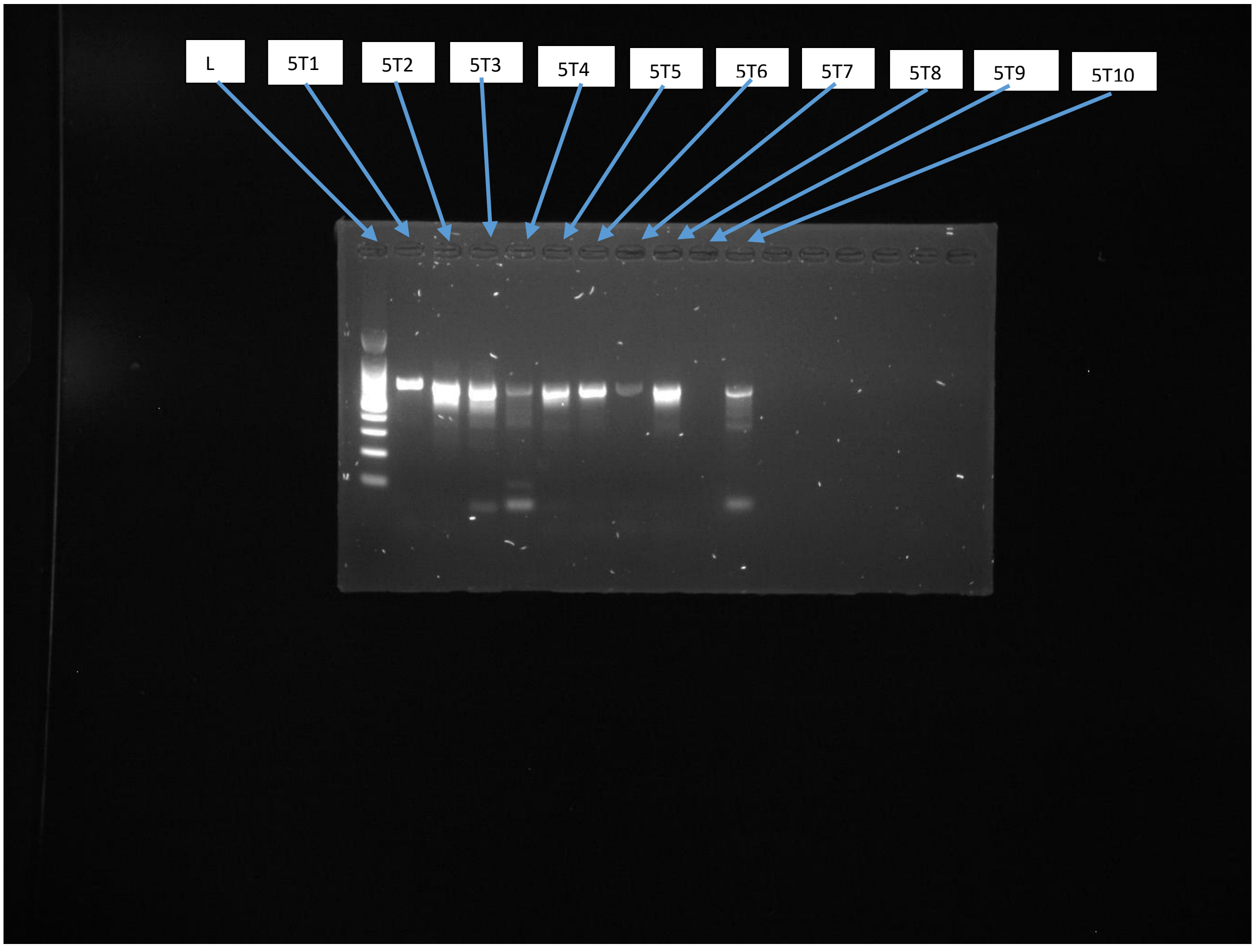

Gel image 5 (612bp): 5T1 to 5T8 fragments amplified

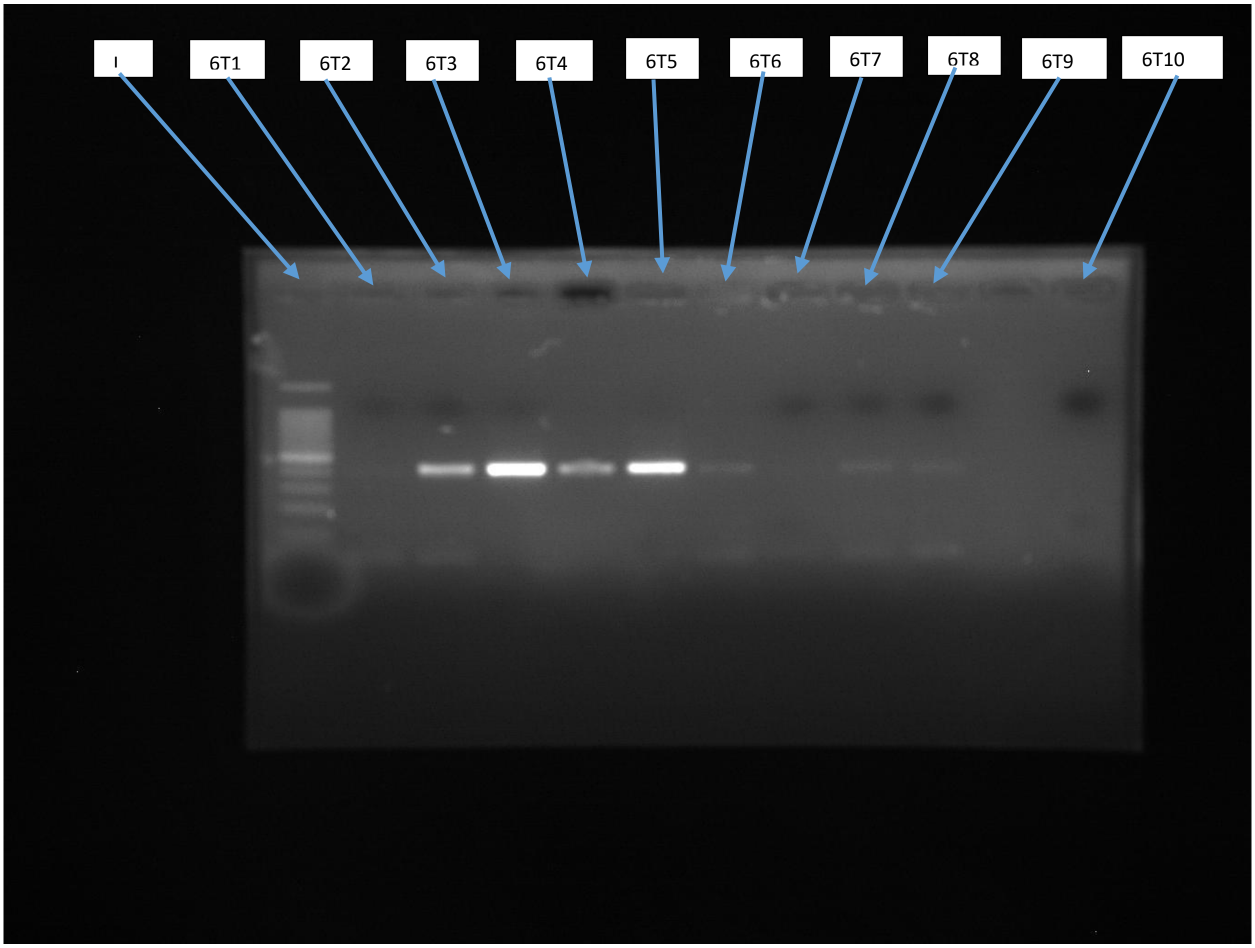

Gel image 6 (445bp): No amplification for 6T1 and 6T7

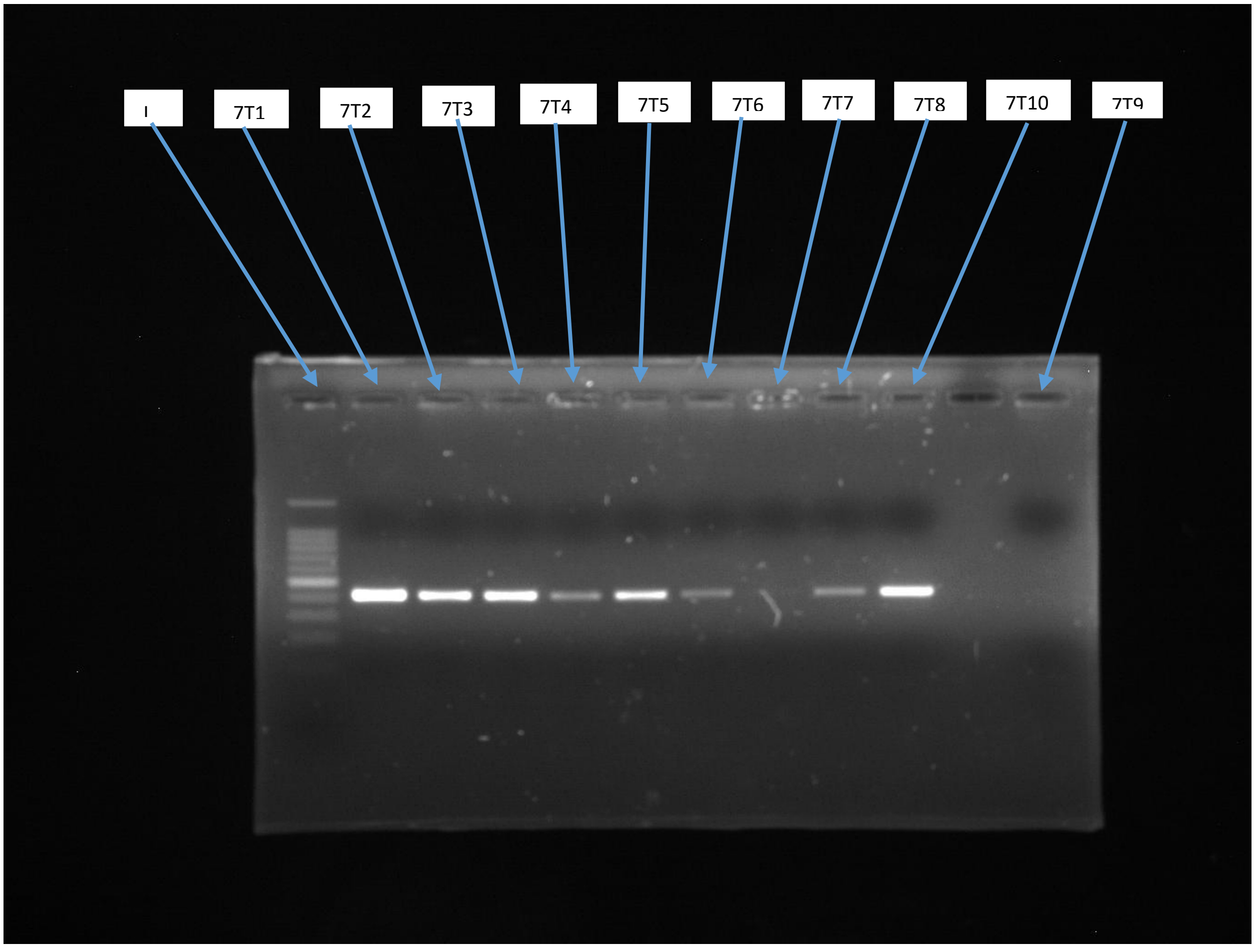

Gel image 7 (395bp): No amplification for 7T7

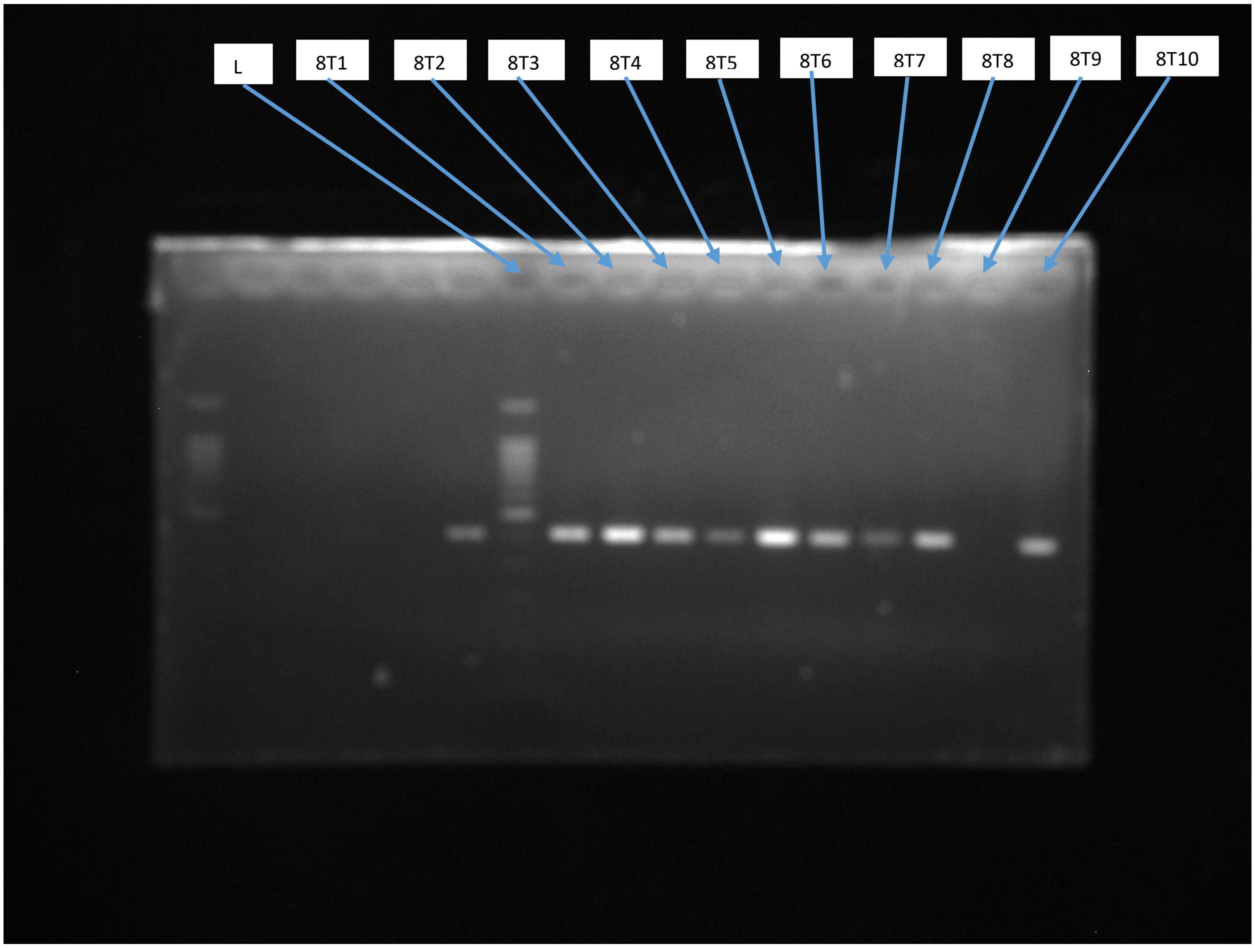

Gel image 8 (389bp): 8T1 to 8T8 all amplified

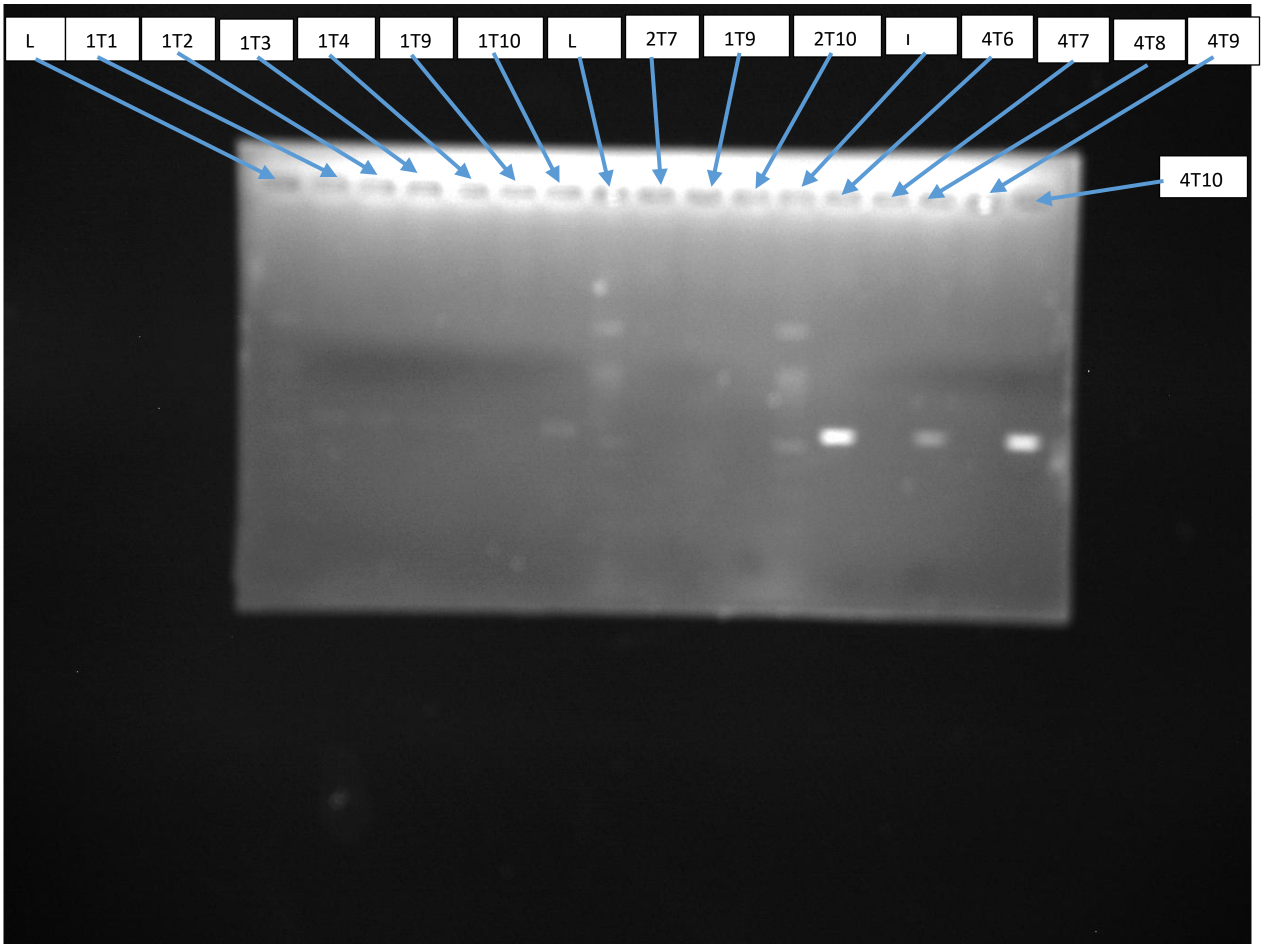

Gel image 1, 2, 4 all repeated: No amplification for 2T7 and 4T7

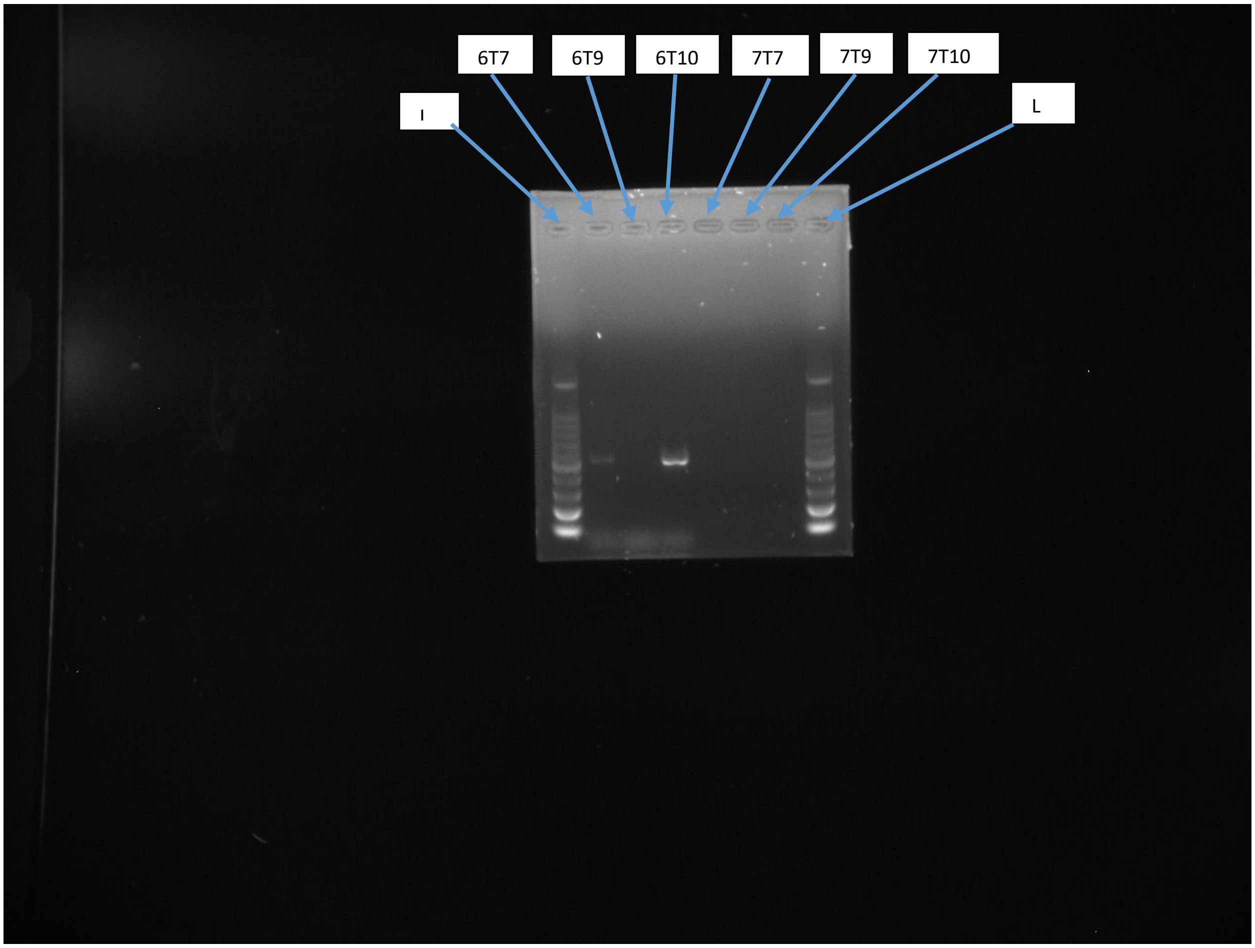

Gel image 6 and 7 repeated for T7: Only 6T7 amplified
