# Supplementary material for: Molecular characterization of haemagglutinin genes of influenza B viruses circulating in Ghana during 2016 and 2017
Source: PLoS One. 2022 Sep 23;17(9):e0271321. doi: 10.1371/journal.pone.0271321 (PMC9506629; doi:10.1371/journal.pone.0271321)
Supplement: Attachment — Submitted filename: Response to Reviewers.pdf [file pone.0271321.s011.pdf]

RESPONSE TO REVIEWERS

Academic Editor
1- Inappropriate funding declaration in manuscript

Corrections made:
All funding-related texts have been removed from the manuscript [Lines 382-384].

Kindly consider replacing the funding statement "The funders had no role in study design, data collection and analysis, decision to publish, or preparation of the manuscript." With the revised funding statement below:

Revised Funding Statement**: "The work was funded by the Armed Forces Health Surveillance Division (AFHSD), Global Emerging Infections Surveillance (GEIS) Branch; PROMIS number P0142_19_N3_08.02.**

2- Request for original uncropped and unadjusted images underlying all blot or gel results report in the submission's figures or supporting information files.

Response:
All original uncropped and unadjusted images have been resubmitted.

Reviewer #1
Major points

1- Are these the only eight sequences positive for influenza B RT-PCR between 2016-2017 from the archive of the study sites? The sample numbers sent to the study sites during 2016-2017 and the samples positive for influenza B viruses during 2016-2017 should be included to calculate the rate of detection. There are no precise inclusion criteria for selecting these eight samples as the representative samples. This information should be included in the materials and methods section.

Response:
No, the eight samples assessed were just a few and they were selected in line with the aim of the study"to identify a path to improve the choice of influenza B vaccine components" and not the description of the circulation of influenza viruses in Ghanain the specific period.
These samples were selected as representatives from the 3 different zones of the country, Southern, Northern and Central Ghana. Specimen positive for Influenza B by real-time RT-PCR during subtyping and with a CT <29 were selected for sequencing and further analysis. Mostly considered because of their strong Ct values upon subtyping and have been mentioned in the materials and methods section [Lines 119-122].

2- The order of figure legends is confusing, starting from Fig. 1, Fig. 4, Fig. 2, Fig. 3.

Response:

The figure legends have been rearranged from Fig. 1, Fig. 2, Fig. 3 and Fig. 4 [Lines 168-179].

3- Materials and Methods: the method/program used for potential glycosylation analysis should be included.

Response:

We only inferred N-glycosylation sites based on the knowledge of the "glycosylationable" amino acid N-X-S/T sequon [Lines 252-260].

4- There is inconsistent information regarding the analysis of antigenic drift. The materials and methods section stated that the analysis was performed for both lineages using the WHO vaccine candidate virus sequences for the 2018-2019 Influenza season. However, the results section mentioned that the sequences were compared with that of the 2016-2017 WHO trivalent and quadrivalent vaccine candidate.

Response:

The 2016-2017 WHO trivalent and quadrivalent vaccine candidates were erroneously written. This has been duly corrected at the results section [Lines 206-207 and 229].

Minor points

1- Materials and Methods: Line 116-117: These had been previously determined to be Influenza B virus positive by real-time RT-PCR using the standard U.S. CDC protocol [15]. -> If the name of the real-time RT-PCR test kit is available, it should be included.

Response:

'QIAGEN One Step RT-PCR kit (QIAGEN, USA)' is inserted in lines 117 and 118.

2- Results: The title that states "Nucleotide changes distinguishing influenza B Victoria from Yamagata lineages" provided no new information. It should be revised to present other important points of the study.

Response:

The new information with respect to the above title is shown in this statement 'The nucleotide changes that distinguish influenza B Victoria from that of influenza B Yamagata lineages from this study support those reported by Arvia et al. (2014), except nucleotide positions 540-542 and 538, which are different' [lines 287 to 289].

3- Results: Line 174: Whole genome amplicon sequencing of the HA genes… should be revised to Targeted sequencing of the full HA genes...

<u>Response</u>:
Whole genome amplicon sequencing of the HA genes… has been replaced with 'Targeted sequencing of the full HA genes….. as recommended [Line 193].

4- Results: S1 Figure should include the size label of the 100 bp marker.

<u>Response</u>:
The 100 bp marker (Ladder) has been labeled appropriately in S1 Figure.

Reviewer #2
1. In the paper "influenza" sometimes it is written with a capital letter, other times with a lowercase. It is necessary to standardize the way of writing.

<u>Response</u>:

Writing of 'influenza' has been duly standardized within the paper except when 'influenza' is beginning with a sentence.

2. In the paper sometimes it is reported B/Victoria and B/Yamagata, other times B Victoria and B Yamagata (with or without /). Also, in this case it is necessary to standardize the way of writing

<u>Response:</u>
Writing of 'B Victoria and B Yamagata' have been duly standardized within the paper.

3. It is unclear whether the samples are collected from 2016-17 or during 2016 and 2017 influenza seasons (line 110)

<u>Response:</u>

Samples were collected during 2016 and 2017 influenza seasons. Revisions have been made to reflect the change – "This retrospective molecular epidemiologic analysis utilized archived influenza B positive clinical samples collected during 2016 and 2017 as part of routine public health surveillance activities of the National Influenza Centre (NIC), which is housed in the Virology Department of the Noguchi Memorial Institute for Medical Research, University of Ghana, Accra." [Lines 109-112].
